# Fixation of Gardens Type 4 Intracapsular Neck of Femur Fracture Using the GS Kulkarni Controlled Dynamic Hip Screw System

**DOI:** 10.7759/cureus.64848

**Published:** 2024-07-18

**Authors:** Mukesh O Phalak, Abhishek Bhadauriya, Archit Gupta, Sagar Gurnani

**Affiliations:** 1 Orthopaedics, Dr. D. Y. Patil Medical College, Hospital and Research Centre, Pune, IND

**Keywords:** controlled dynamic hip screw, fracture femoral neck, dynamic hip screw fixation, fixation, femoral neck fracture

## Abstract

The goal for surgical treatment of femoral neck fractures is achieving union through an anatomic reduction and stable fixation while avoiding osteonecrosis. This report describes the case of a 50-year-old male who presented with pain and swelling in his right hip and inability to bear weight over his right lower limb following a road traffic accident. The X-ray revealed a right-sided femoral neck fracture (Garden Type IV and Pauwels Type III). The patient was surgically treated with the GS Kulkarni (GSK) controlled dynamic hip screw. On follow-up, the bone healing time was 14 weeks and the patient achieved a Harris hip score of 93.65 with significant improvement.

## Introduction

Intracapsular femoral neck fractures affect mostly the elderly population. Patients from younger age group are substantially less likely to experience femoral neck fractures, which often originate from high-energy mechanisms. The medial femoral circumflex's terminal branches are intracapsular and at risk of being damaged (kinked or torn) by the displacement of femoral neck fractures, which may play a crucial part in the onset of osteonecrosis [1,2].

Femoral neck fractures occur intra-synovially; the cambium layer is absent in the periosteum. As a result, subsequent fracture healing is not promoted. Instead, only direct osteonal remodeling can cure fractures. An anatomic reduction and compression are necessary for this kind of bone repair. Primary healing may occur if there is a minor gap in the gap healing procedure. These elements play a part in the high reported rates of avascular necrosis (10-23%), non-union (8-19%), malunion (7.1%), implant failure (9.7%), and requirement for reoperation (20%) [2]. Surgery for femoral neck fractures in young patients aims to achieve union through anatomic reduction and stable fixation while preventing osteonecrosis.

The timing of the surgery is still debatable. The benefits of early surgery include vessel unkinking through prompt reduction and an intracapsular decompression, both of which aim to enhance femoral head perfusion and lower the risk of osteonecrosis. This strategy is supported by research that found that delayed treatment increases the likelihood of osteonecrosis. Contrarily, several studies have found no change in the rate of osteonecrosis following surgical therapy that was delayed for longer than 24 hours. Internal fixation surgical techniques frequently employed include the application of hollow compression screws, dynamic hip screws, and compression plate(s) [3]. In a study by Parker and Stedtfeld on intracapsular neck of femur fracture, analysis of the healed fractures reveals a mean of 7 mm of TeleScrew lateral displacement at the fracture site for the un-displaced fractures and a mean of 12 mm of lateral displacement at the fracture site for the displaced fractures. The collapse at the fracture spot was the cause of this slide [4].

In this case, we have used a GS Kulkarni (GSK) controlled dynamic hip screw (DHS), which is a DHS system that allows for controlled/limited collapse at the fracture site, for fixation of a Gardens type IV intracapsular neck of femur fracture and evaluated the clinical and functional outcome of the same [5]. Dr GS Kulkarni one of the pioneers of DHS and the GSK controlled DHS allows for controlled collapse at the fracture site with variable amounts of slide or lateral displacement.

## Case presentation

A 50-year-old male patient presented to the emergency department of our hospital with complaints of pain and swelling in the right hip and inability to bear weight over the right lower limb following a road traffic accident four hours prior to presentation. Distal pulsations and sensations were intact in the injured lower limb. There was no other associated injury and the patient was vitally stable.

**Figure 1:**
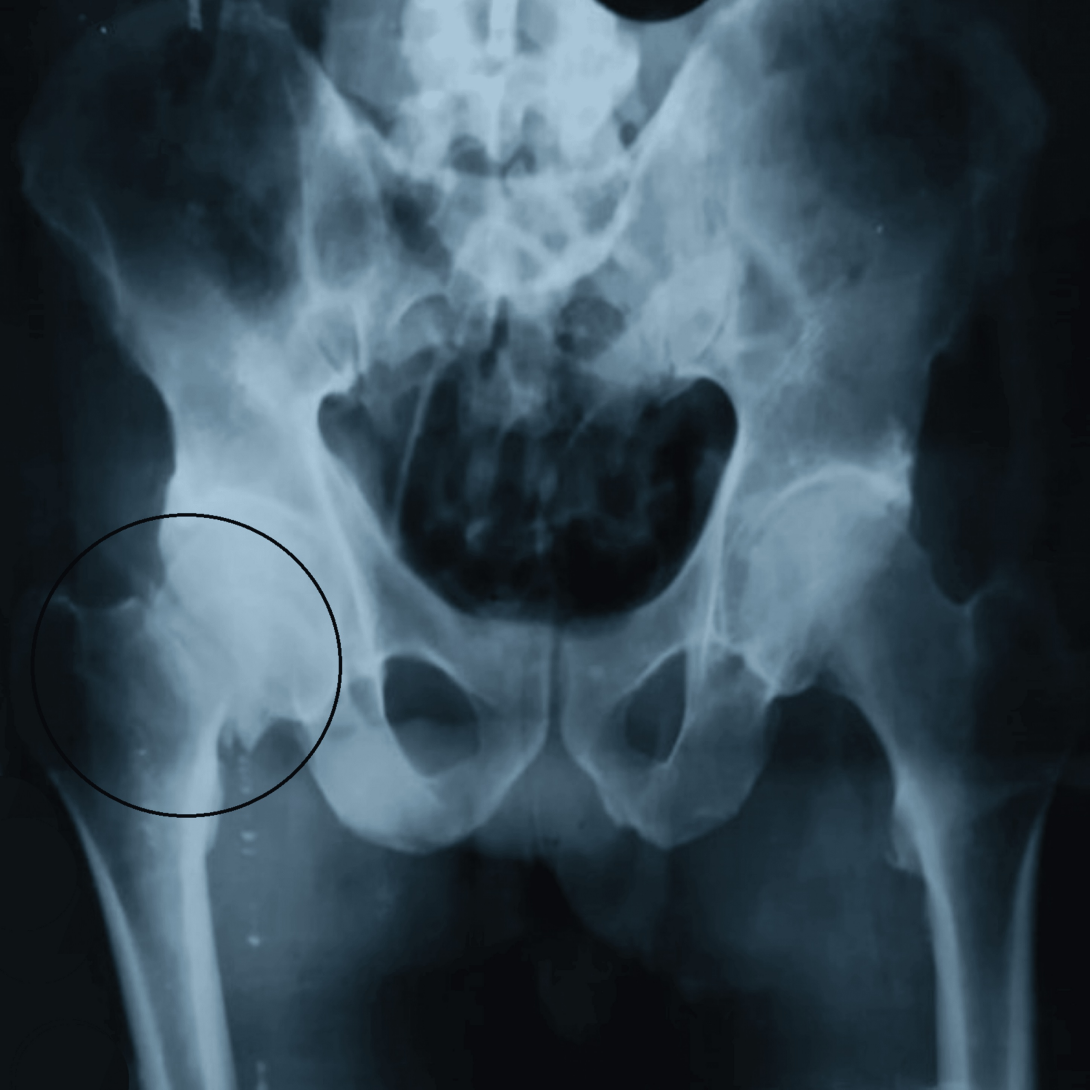
X-ray of pelvis with both hips (anteroposterior) showing right-sided femoral neck fracture (Garden Type IV and Pauwels Type III)

**Figure 2:**
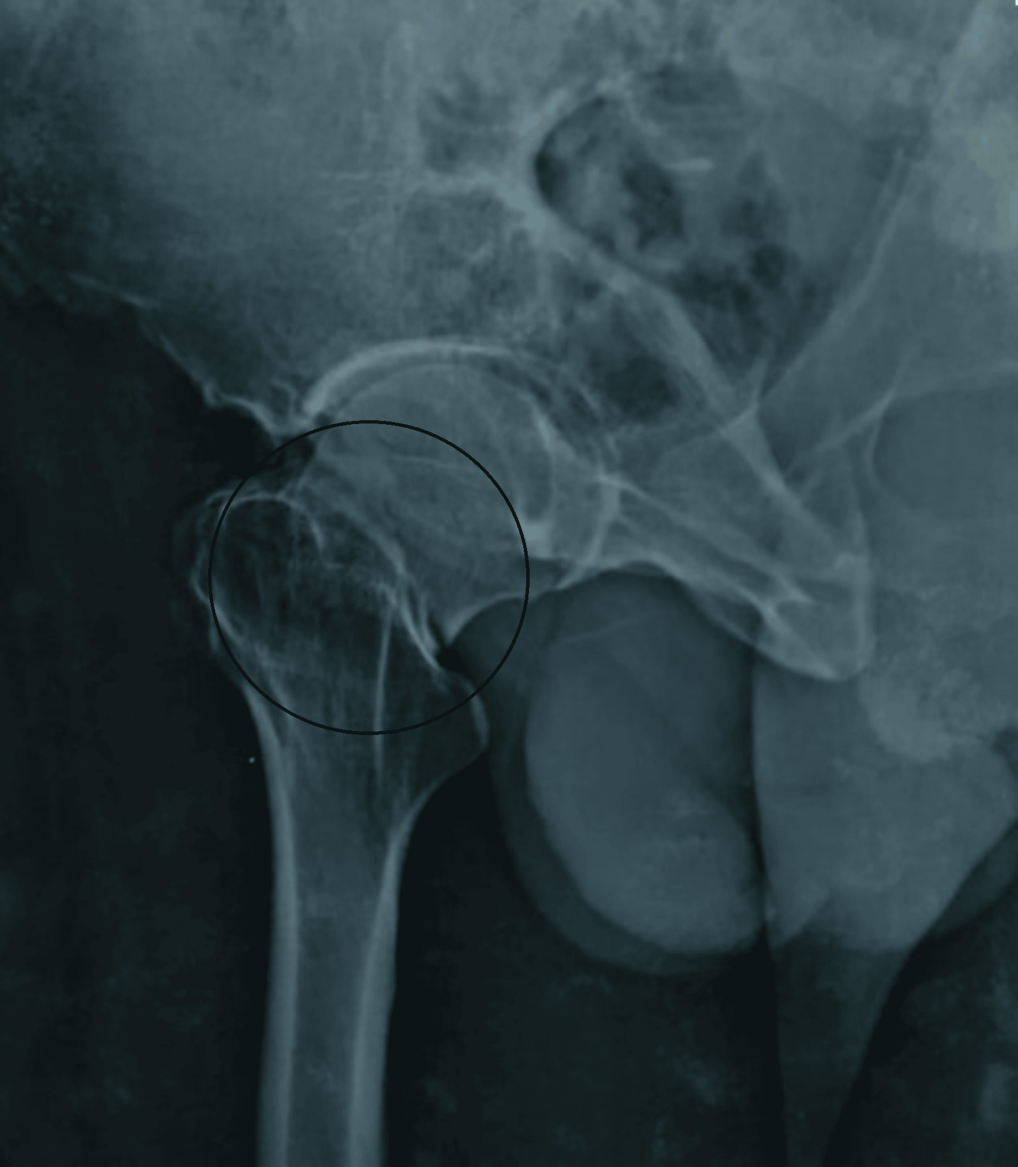
X-ray of the right hip (cross-table lateral) showing right-sided femoral neck fracture (Garden Type IV and Pauwels Type III)

Plain X-rays of the pelvis with both hips (anteroposterior) and right hip (cross-table lateral) along with all routine investigations. The X-rays revealed a right-sided femoral neck fracture (Garden Type IV and Pauwels Type III) shown in Figures 1, 2.

Plan for surgery

The patient was planned to undergo open reduction and internal fixation (ORIF) with DHS (GSK-controlled DHS) fixation under spinal anesthesia. In DHS, the sliding mechanism causes excessive collapse of the head which leads to complications like non-union, avascular necrosis (AVN), and femoral neck shortening which causes limb shortening, changes in the biomechanics of hip and abductor weakness, and lurch. In GSK-controlled DHS, there is a provision for controlled collapse allowing for collapse at the fracture site in the range of 1-10 mm. There are options for three top screws; the conventional top screw allows a sliding of 1 mm, the 5 mm head top screw allows for a sliding of 10 mm, and the 10 mm head top screw allows for a sliding of 5 mm. The top screw is selected as per the desired collapse needed at the fracture site.

Surgical procedure

**Figure 3:**
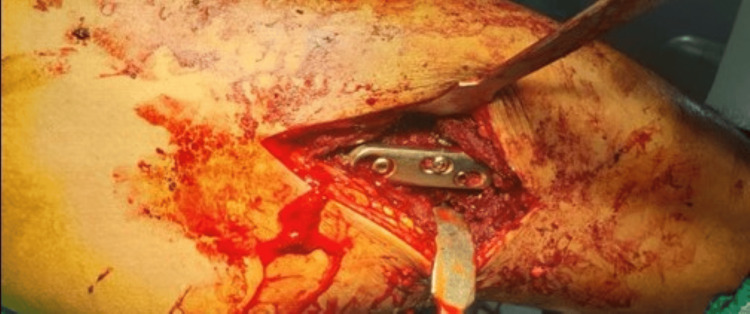
Clinical view of the surgical procedure showing the DHS in situ DHS: dynamic hip screw

**Figure 4:**
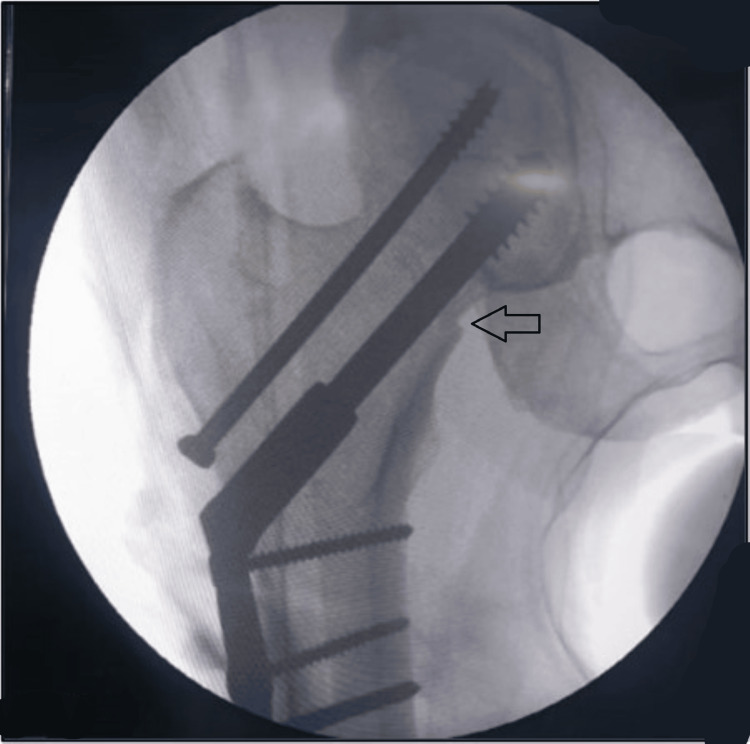
Radiological view (anteroposterior) of the surgical procedure showing the hip joint with implant in situ Arrow showing adequate compression at the fracture site

**Figure 5:**
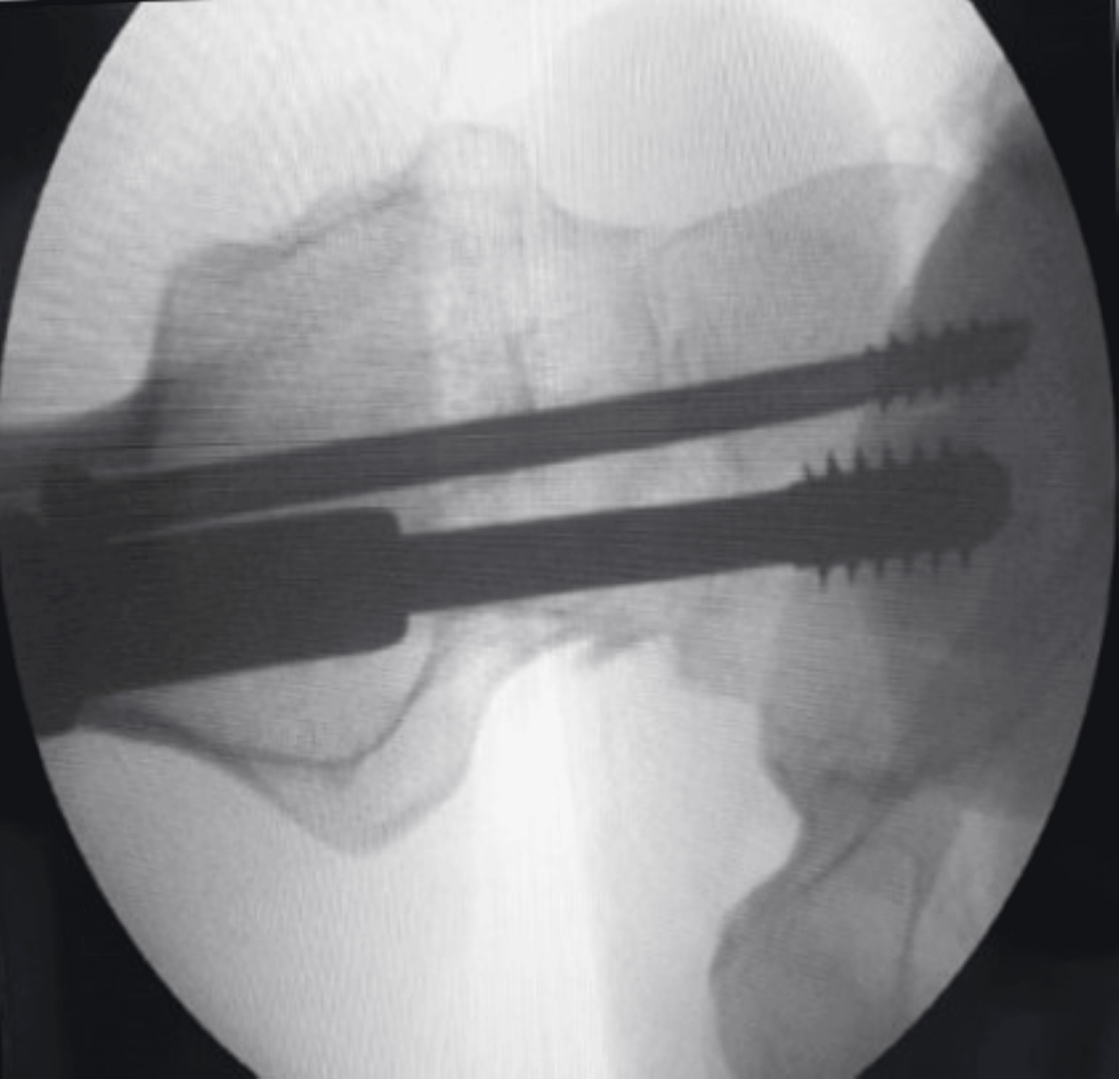
Radiological view (lateral) of the surgical procedure showing the neck of femur

**Figure 6:**
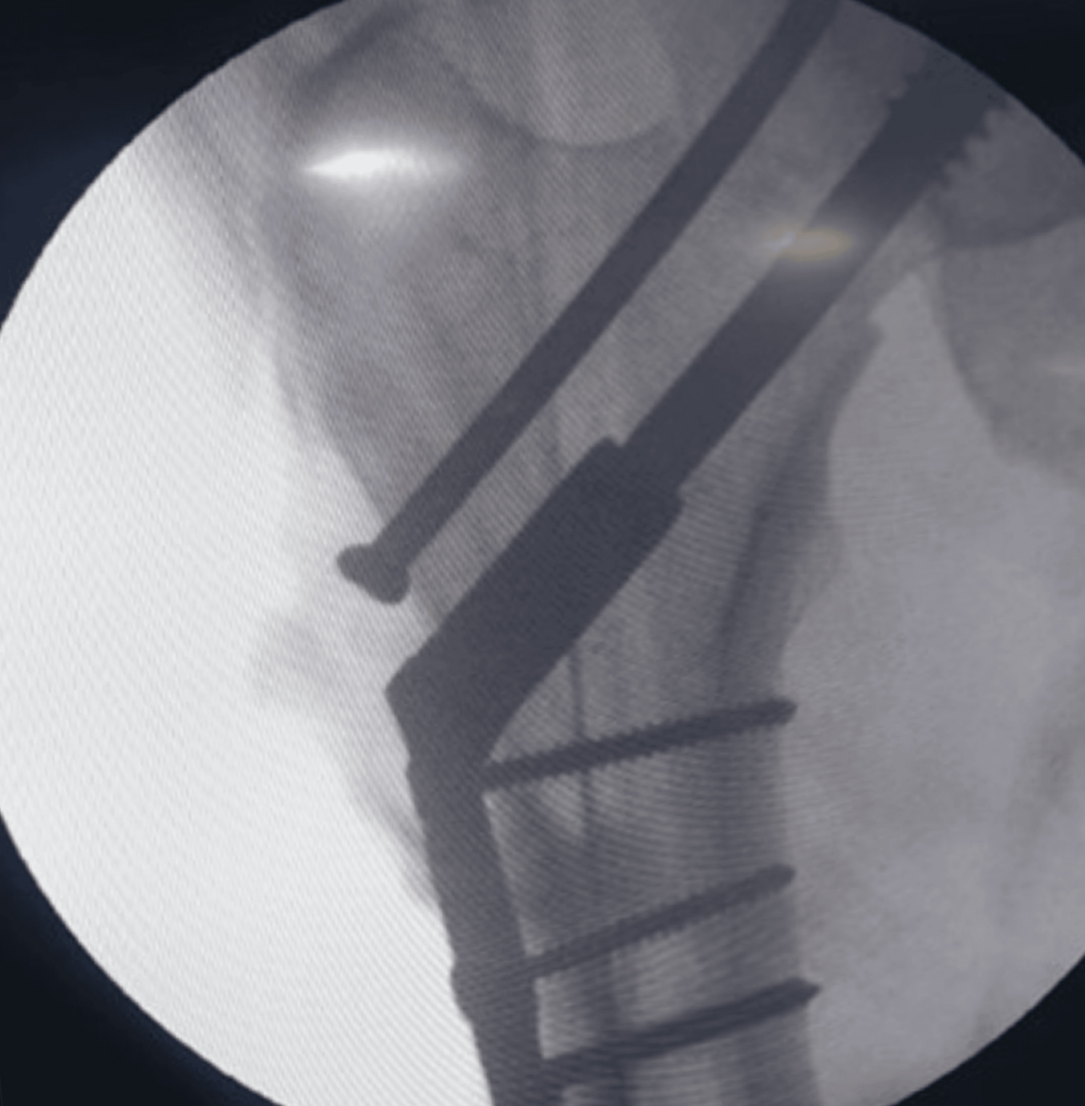
Radiological view of the surgical procedure

The patient was taken on an orthopaedic fracture table after spinal anesthesia. The limb was kept in abduction and internal rotation. The reduction was achieved after traction and manipulation with the help of the fracture table and confirmed under an X-ray image intensifier. With all sterile precautions, an approximately 6 cm long incision was made under the greater trochanter extending distally over the shaft of the femur. Subsequently, the lateral femoral surface was exposed for satisfactory hardware placement. A guide wire for a 6.5 mm CC screw was put in the upper part of the neck followed by a guidewire for the DHS in the inferior part of the neck and confirmed under an X-ray image intensifier. Then, the 6.5 mm de-rotation screw was put in. Then the triple reamer for DHS was put over the DHS guide wire and reaming was done followed by tapping. A lag screw of 85 mm was passed over the DHS guide wire. A DHS three-hole plate was placed over the proximal femur and the barrel was slid over the lag screw. The distalmost screw of the DHS plate was put in to fix the plate with the femur shaft. After removing the DHS guide wire, a 10 mm top screw was put in the lag screw, which allowed for sliding of 5 mm at the fracture site. De-collapsing screw was put just distal to the top screw. Finally, the remaining second screw of the DHS plate was put in (Figure 3). The final reduction was confirmed under the X-ray image intensifier (Figures 4-6). A thorough wash was given and closure was done in layers.

Postoperative protocol

**Figure 7:**
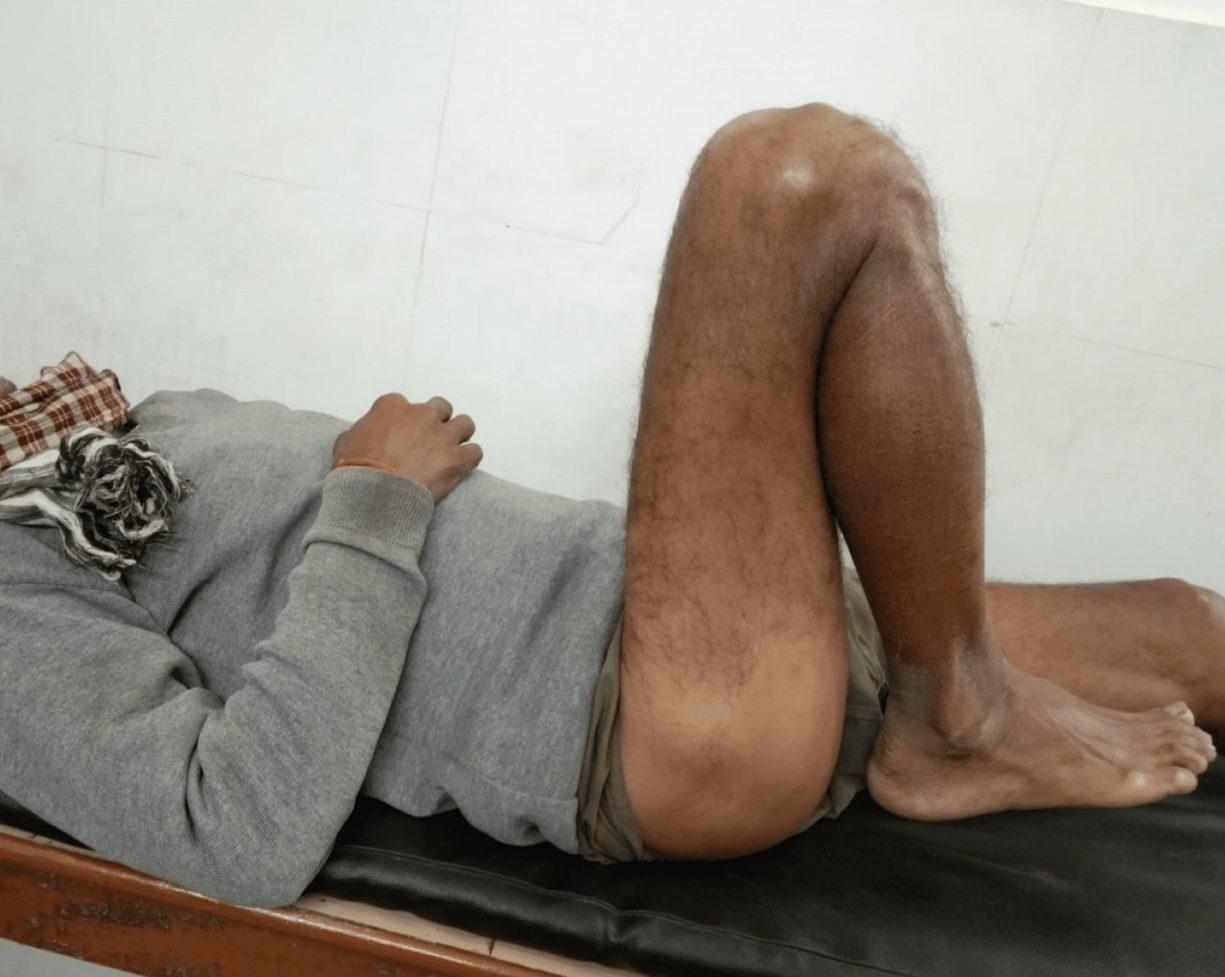
Clinical view of the postoperative follow-up showing full flexion at the hip joint

**Figure 8:**
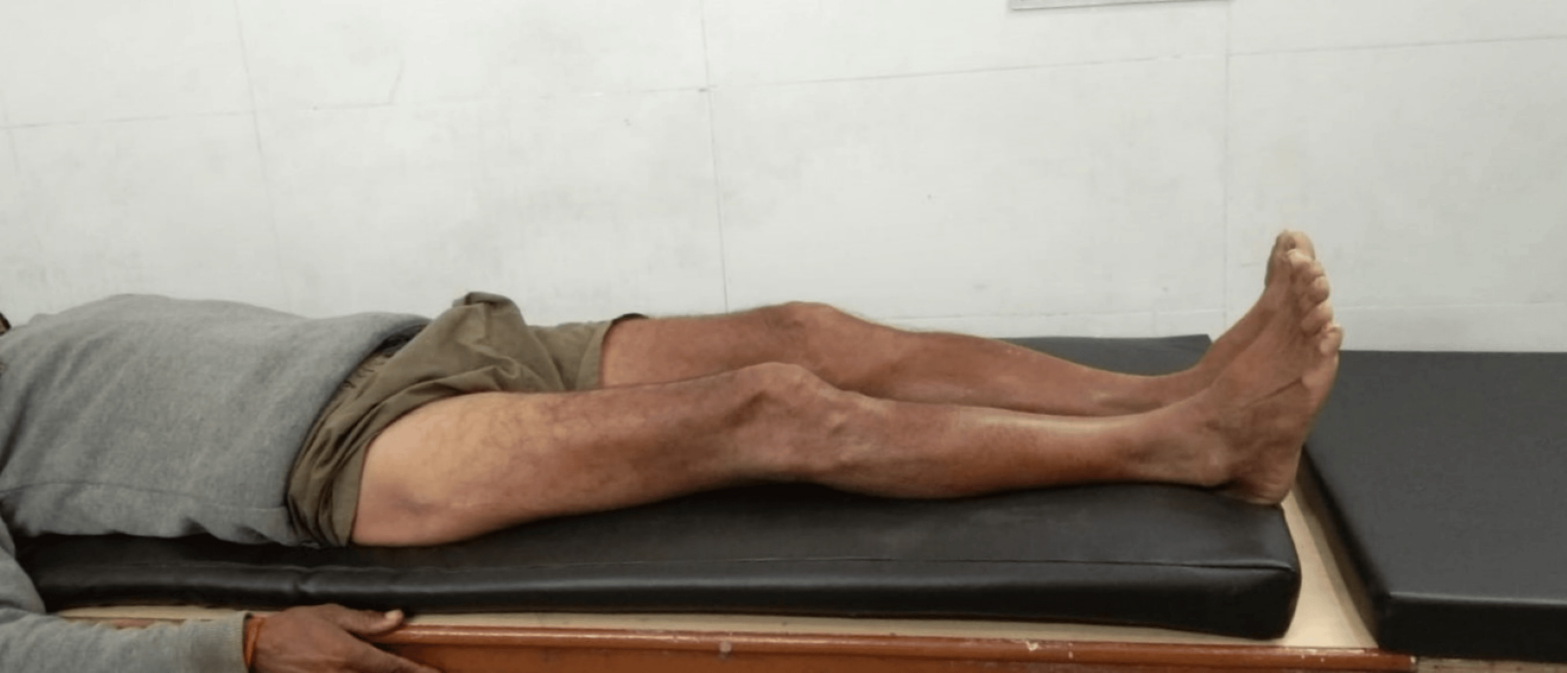
Clinical view of the postoperative follow-up showing no limb length discrepancy

**Figure 9:**
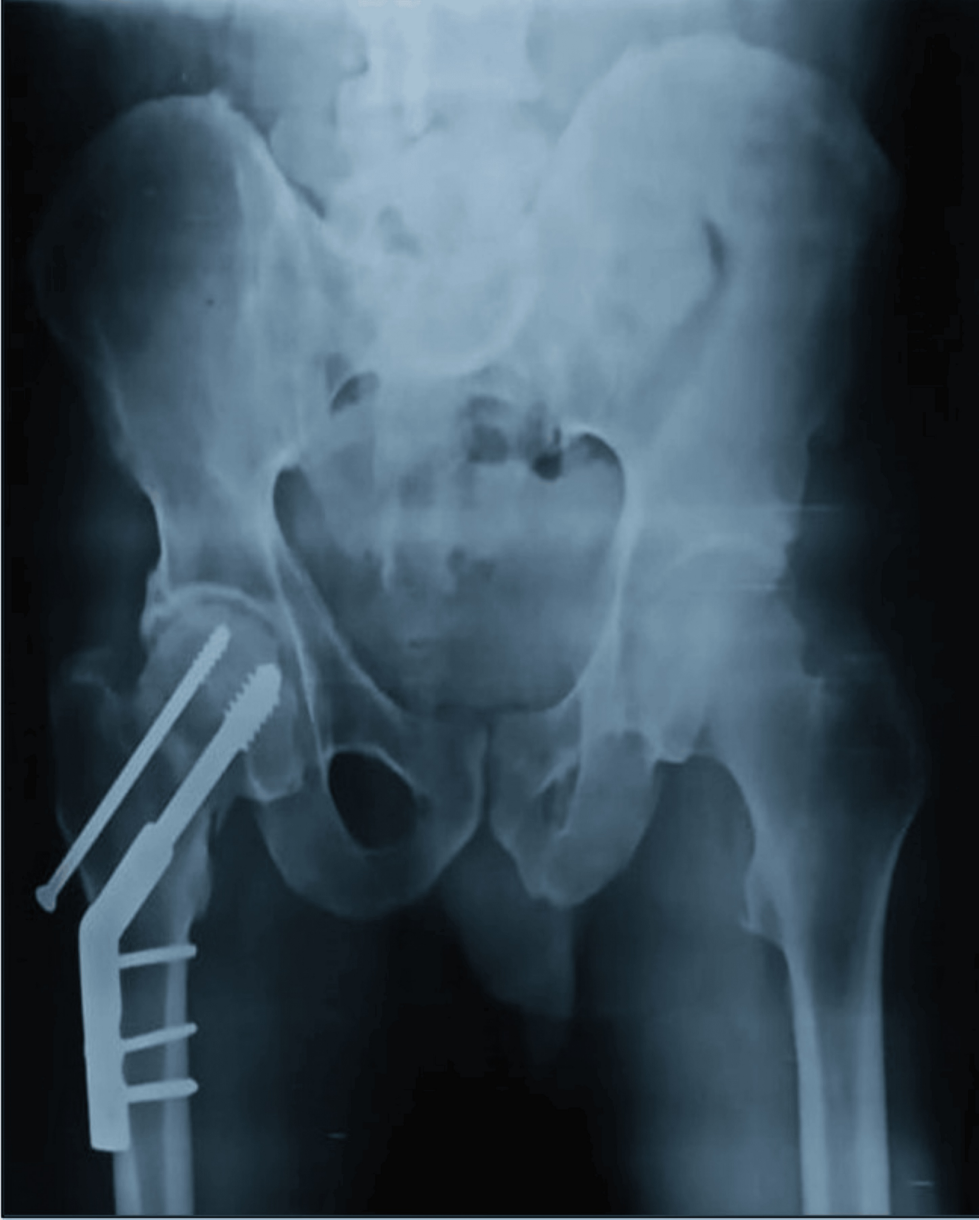
Radiological view of the postoperative follow-up showing pelvis with both hips

**Figure 10:**
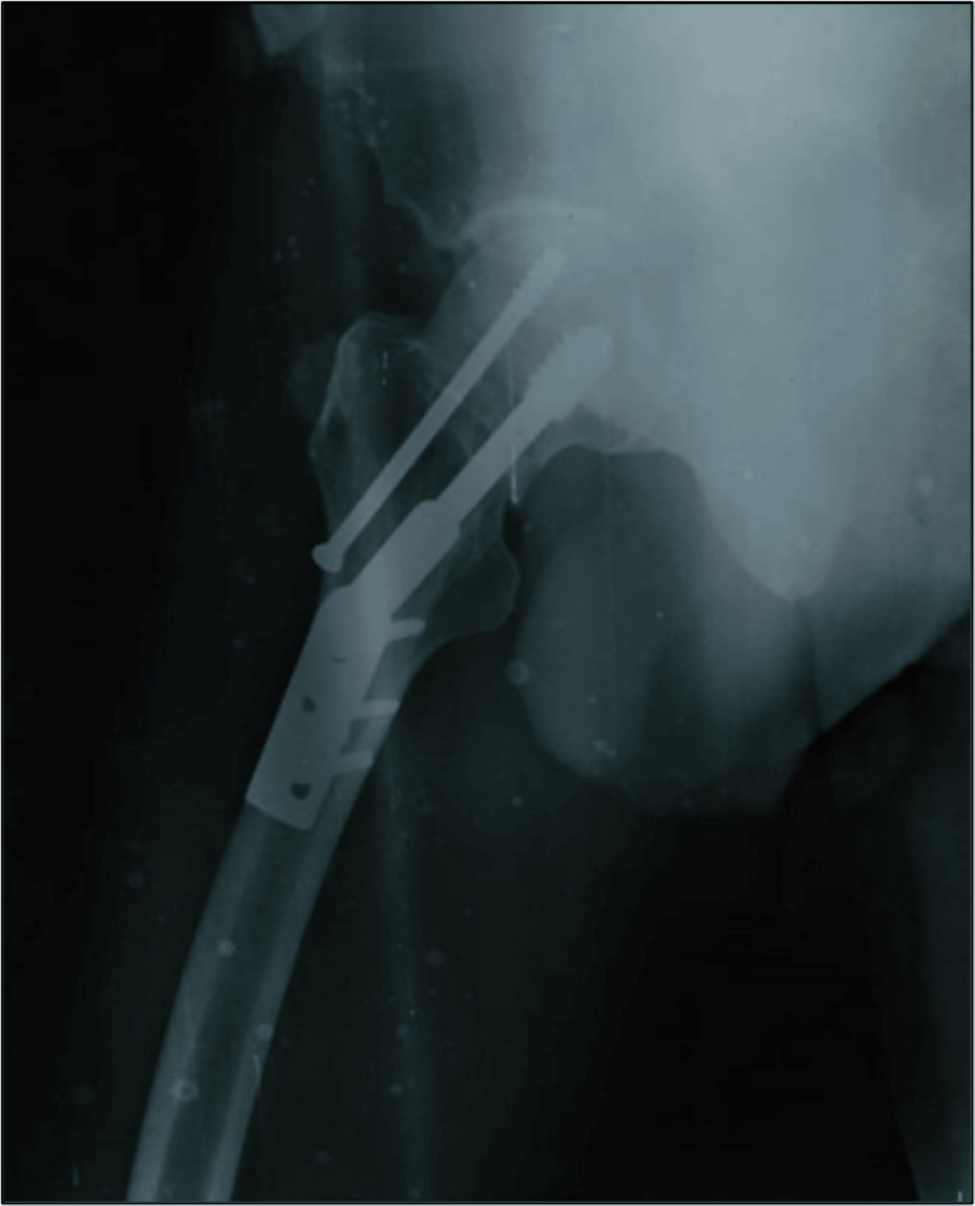
Radiological view at the postoperative follow-up

The patient was started on hip range-of-motion (ROM) exercises on postoperative day 1 of surgery and gradually increased as tolerated by the patient. The patient was kept strictly non-weight-bearing for three weeks and mobilized with the help of a walker. After three weeks, the patient was started on partial weight-bearing for the next three weeks. After six weeks of follow-up, the patient was started on full weight-bearing walking. Full ROM was achieved as seen in Figure 7. No limb length discrepancy was present as evident in Figure 8. Postoperative follow-up radiographs are shown in Figures 9, 10, which show satisfactory union. 

## Discussion

This study investigated the clinical and functional outcomes achieved by treating femoral neck fractures with GSK-controlled DHS. Samsami et al. found that DHS in combination with an anti-rotation screw was the biomechanically more effective technique for fixation [6].

Weil reported that 22% of patients had significant shortening (more than 10 mm) and that 56% of patients had femoral neck shortening (greater than 5 mm) [7]. Seventeen (41%) patients experienced screw backing out (more than 5 mm). Seven patients required arthroplasty that was performed more than six months after the first internal fixation. Age, neck shaft angle, fracture type, or reduction quality had no statistically significant relationship with femoral neck shortening.

In a study by Felton et al., it was found that two-thirds of patients showed no or mild shortening (5 mm) and the remaining one-third showed moderate or severe shortening (>5 mm) [8]. They reported that the hip function was deteriorating as shortening increased. Slobogean et al. observed that at 12 months following fixation, individuals under the age of 55 with femoral neck shortening >10 mm had a mean Harris Hip Score 9.9 points lower than those with less shortening [9]. Similar findings were established by Zlowodzki et al. who showed that following internal fixation with cancellous screws for undisplaced femoral neck fractures, physical functioning scores dropped with the increase in shortening [10]. They reported variations in scores based on the degree of shortening, with a higher degree of shortening demonstrating poorer functional outcomes.

He et al. reported that femoral neck shortening was observed in one of the patients in the femoral neck system group and in three patients in the cannulated screw group [3]. One patient from the femoral neck system group and two from the cannulated screw group experienced cut-out in internal fixation. Two months following surgery, one patient in the cannulated screw group had their nails removed, two patients experienced bone nonunion, and there was no sign of callus growth at the fracture site. The remaining patients in both groups experienced clinical healing of the bones.

Cannulated compression screws and the femoral neck system were evaluated as two distinct internal fixation techniques for the treatment of femoral neck fractures in patients under the age of 60 in a study by Hu et al. [11]. The femoral neck system group's Harris Hip Scores were marginally greater and the bone healing process took place far faster than the cannulated compression screw group. Both groups experienced a shortened femoral neck following surgery. The femoral neck system group experienced much less femoral neck shortening than the cannulated compression screw group. No marked difference was found between the two groups in terms of occurrence of femoral head necrosis or fracture nonunion following the surgery. A considerably lower incidence of femoral neck shortening and screw cut-out was observed in the femoral neck system group.

In our study, we used GSK controlled DHS for the fixation of a Gardens classification Type IV and Pauwels classification Type III femoral neck fracture in a 50-year-old male patient. The bone healing time in our patient was 14 weeks and the patient achieved a Harris Hip Score of 93.65 with significant improvement.

## Conclusions

The rate of revision surgery following internal repair of femoral neck fractures is high under current treatment options. Limited clinical data show that a high percentage of patients heal with the femoral neck in a shortened position, which is detrimental to the patient's physical function. The application of GSK-controlled DHS for the fixation of a Gardens classification Type IV and Pauwels classification Type III femoral neck fracture showed promising outcomes. Further research on this method in a large population with a comparative approach with commonly used methods is recommended.
